# *Chlorella sorokiniana* Extract Prevents Cisplatin-Induced Myelotoxicity *In Vitro* and *In Vivo*

**DOI:** 10.1155/2020/7353618

**Published:** 2020-01-25

**Authors:** Shyh-Horng Lin, Ming-Han Li, Kai-An Chuang, Ni-Hsuan Lin, Chih-Hsuan Chang, Hsin-Chieh Wu, Ya-Hsuan Chao, Chi-Chien Lin, I-Hong Pan, Ming-Der Perng, Shu-Fang Wen

**Affiliations:** ^1^Biomedical Technology and Device Research Laboratories, Industrial Technology Research Institute, 321 Kuang Fu 2^nd^ Road, Hsinchu 30011, Taiwan; ^2^Institute of Molecular Medicine, College of Life Sciences, National Tsing Hua University, 101 Kuang Fu 2^nd^ Road, Hsinchu 30013, Taiwan; ^3^Institute of Biomedical Science, National Chung-Hsing University, 145 Xingda Road, Taichung 40227, Taiwan

## Abstract

Cisplatin chemotherapy causes myelosuppression and often limits treatment duration and dose escalation in patients. Novel approaches to circumvent or lessen myelotoxicity may improve clinical outcome and quality of life in these patients. *Chlorella sorokiniana* (CS) is a freshwater unicellular green alga and exhibits encouraging efficacy in immunomodulation and anticancer in preclinical studies. However, the efficacy of CS on chemoprotection remains unclear. We report here, for the first time, that CS extract (CSE) could protect normal myeloid cells and PBMCs from cisplatin toxicity. Also, cisplatin-induced apoptosis in HL-60 cells was rescued through reservation of mitochondrial function, inhibition of cytochrome c release to cytosol, and suppression of caspase and PARP activation. Intriguingly, cotreatment of CSE attenuated cisplatin-evoked hypocellularity of bone marrow in mice. Furthermore, we observed the enhancement of CSF-GM activity in bone marrow and spleen in mice administered CSE and cisplatin, along with increased CD11b levels in spleen. In conclusion, we uncovered a novel mechanism of CSE on myeloprotection, whereby potentially supports the use of CSE as a chemoprotector against cisplatin-induced bone marrow toxicity. Further clinical investigation of CSE in combination with cisplatin is warranted.

## 1. Introduction

Chemotherapy is the most effective and widely used treatment in most types of cancers [1]. Of which, cisplatin, cis-diamminedichloroplatinum(II), has been used over 40 years for treating at least 18 distinct tumor types as monotherapy or combination therapy with other chemotherapeutics, radiation therapy, and/or surgery, albeit lack of the cellular and molecular mechanisms that underlie its efficacy [2, 3]. Indeed, cisplatin is the standard of care in children for treatment of hematological tumors and in adults for treatment of solid tumors such as testicular, prostate, urothelial, ovarian, cervical, breast, brain, bladder, esophageal, head and neck cancers, and nonsmall and small-cell lung cancer. The antitumor efficacy of cisplatin primarily cross-links with DNA and subsequently interferes with DNA transcription and/or DNA replication [4]. However, cisplatin is associated with several adverse effects in patients, including renal, neuronal, auditory, bone marrow, and gastrointestinal toxicities (e.g., nausea and vomiting) [5]. Often, these toxic effects give rise to subtherapeutic dose delivery and/or discontinuation of chemotherapy, ultimately compromise treatment outcomes such as disease control and survival in patients with curable malignancies [6]. In particular, myelotoxicity is closely related to morbidity, mortality, cost, and reduced chemotherapy dose intensity and treatment failure [7]. Therefore, it has received great attention in developing novel chemoprotective agents to reduce the overall toxicity associated with cisplatin.

A chemoprotective agent that alleviates the adverse effects of cisplatin without affecting its therapeutic effect would definitely have clinical benefit. Although several natural and synthetic compounds have been reported to be chemoprotective, such as hydrogen sulfide [8], vitamins C [9], resveratrol, and genistein [10], the only FDA approved and commonly accepted chemoprotective drug for cisplatin therapy is amifostine, which is a sulfur-containing agent that reduces renal toxicity and neutropenia caused by different chemotherapy and radiotherapy regimens [11]. However, it might diminish cisplatin's activity and may lessen the efficacy of cisplatin [12]. In addition, amifostine by itself is related to apparent side effects, including hypotension, nausea, and vomiting [13]. Therefore, there is a high demand in finding significantly improved chemoprotectors against cisplatin-induced toxicities.

In the present study, we investigated the chemoprotective ability and molecular mechanisms of *Chlorella sorokiniana* against cisplatin toxicity *in vitro* and evaluated antimyelotoxicity effects of *Chlorella sorokiniana in vivo*. *Chlorella sorokiniana* is a species of *Chlorella*, a genus of freshwater unicellular green algae [14]. The extracts of *Chlorella* have been demonstrated for potentially improving human health and wildly used as botanical foods in modulation of human immune responses [15, 16]. Importantly, *Chlorella* extracts possess various beneficial pharmacological effects against cancers [17], bacterial infections [18], and viral replication [19]. From an earlier study, *Chlorella* extract was reported to strongly increase the production of IFN-*γ* and IL-2 and activate Th1 cells to strengthen the immune system and host defense [20]. Along with this, *Chlorella sorokiniana* was found to exhibit immunomodulatory effects in human monocyte-derived dendritic cells through NF-*κ*B and PI3K/MAPK pathways [21]. Most recently, Lin et al. revealed *Chlorella sorokiniana* exerts effects on inhibiting xenograft tumor growth and inducing mitochondria-mediated apoptosis in human non-small-cell lung cancer cells [22]. Despite the fact that *Chlorella sorokiniana* is involved in the anticancer and immunomodulatory bioactivities, it is not clear whether *Chlorella sorokiniana* can reduce the toxicity resulted from chemotherapeutic drugs. Here, we report the effectiveness of *Chlorella sorokiniana* in the prevention of cisplatin-induced toxicity. We have shown that *Chlorella sorokiniana* prevents cisplatin-induced apoptosis in myeloid cells through a mitochondrial-dependent caspase activation pathway. Also, *Chlorella sorokiniana* was able to reduce bone marrow toxicity in mice upon cisplatin exposure. Thus, our results identify a novel chemoprotective role of *Chlorella sorokiniana* in the prevention of cisplatin-induced toxicity and suggest that this natural product could be developed as a chemoprotective agent in cancer therapy.

## 2. Materials and Methods

### 2.1. Reagents and Chemicals

Liquid form of *Chlorella sorokiniana* extract (CSE) was provided by International Cryptomonadales Biotechnology (W87; Changhua, Taiwan). The *Chlorella sorokiniana* W87 was refluxed with purified water for 1 h, and the algae residue was removed by a high speed separator and concentrated at 60°C until the solid content of liquid extract was 5%. Cisplatin was purchased from Fresenius Kabi Oncology (Haryana, India). Other reagents and chemicals were obtained from Sigma-Aldrich (St. Louis, MO, USA) unless otherwise specified.

### 2.2. Cell Culture

HL-60 (human promyelocytic leukemia cell line) and THP-1 (human acute monocytic leukemia cell line) were obtained from the American Type Culture Collection and cultured at 37°C in a humidified atmosphere of 5% CO_2_ and 95% air. The HL-60 cells were incubated in Iscove's Modified Dulbecco's Medium (IMDM) supplemented with 20% fetal bovine serum (FBS; Thermo Fisher Scientific, Waltham, MA USA), 50 U/mL penicillin, 50 *μ*g/mL streptomycin (Thermo Fisher Scientific), 25 mM HEPES, and 2 mM L-glutamine (both from Invitrogen, Carlsbad, CA). THP-1 cells were maintained in RPMI-1640 containing 10% FBS, 10 mM HEPES, 1 mM sodium pyruvate, 4.5 g/L glucose, 1.5 g/L sodium bicarbonate, 50 U/mL penicillin, 50 *μ*g/mL streptomycin, and 2 mM L-glutamine.

### 2.3. Measurement of Cytotoxicity

The HL-60 or THP-1 (2 × 10^4^ cells per well) were seeded into 96-well plates for 16 h and then treated with cisplatin and CSE for 72 h. Subsequently, the Alamar Blue assay (AbD Serotec, Raleigh, NC, USA) was carried out (10% (*v*/*v*), 37°C, 4 h) to evaluate cell viability. The absorbance was measured at wavelengths of 570 nm (oxidized state) and 600 nm (reduced state) using a microplate spectrophotometer (SpectraMax M5, Molecular Devices, USA). Cell viability was calculated as the mean percentage relative to untreated cells.

### 2.4. Flow Cytometric Analysis

Cellular apoptosis was detected with a FITC Annexin V Apoptosis Detection Kit (BD Biosciences, San Jose, CA, USA) following the manufacturer's instructions. After harvesting, HL-60 and THP-1 cells were spun down in Eppendorf tubes and resuspended in 1x binding buffer, after which 5 *μ*L of Annexin V/PI or a buffer control was added according to the manufacturer's instructions. Next, flow cytometric analysis was performed on a CyFlow space instrument (Partec, Münster, Germany). The resulting data were analyzed using FloMax software (Partec).

### 2.5. Mitochondrial Fractionation

Mitochondrial-enriched fractions were prepared according to a previously published protocol [23]. Briefly, cells were homogenized on ice in IB-1 buffer (225 mM mannitol, 75 mM sucrose, 1 mM EDTA, 10 mM HEPES; pH 7.4), and the total homogenate was centrifuged at 600 ×*g* at 4°C for 10 min. The supernatant was further centrifuged at 7,000 ×*g* at 4°C for 10 min, and the resulting supernatant was collected as the cytosolic fraction. The remaining pellet, representing the mitochondrial-enriched fraction, was resuspended in IB-2 buffer (225 mM mannitol, 75 mM sucrose, 20 mM HEPES; pH 7.4) and centrifuged at 9,000 ×*g* for 10 min at 4°C. The final pellet was resuspended in sample buffer (25 mM Tris-HCl, pH 6.8, 5 mM EGTA, 1% (*w*/*v*) SDS) and sonicated for 30 sec prior to analysis by immunoblotting.

### 2.6. Western Blot Analysis

HL-60 cells were seeded in 6 cm petri dishes at a density of 1 × 10^6^ and cultured for 24 h. Then, cisplatin and CSE were added and the cells were allowed to culture for 48 h. Western blot analysis was conducted as previously described [24]. Proteins were extracted from the cells using radioimmunoprecipitation assay (RIPA) lysis buffer supplemented with 1 mM phenylmethylsulfonyl fluoride (PMSF), a cocktail of protease inhibitors (Roche, Mannheim, Germany), and phosphatase inhibitors (Merck Millipore, Billerica, MA, USA). The samples were separated on a NuPAGE 4-12% Bis-Tris gel (Thermo Fisher Scientific) before transferring to a polyvinylidene difluoride membrane (Amersham Biosciences, Piscataway, NJ, USA) using the wet electrophoretic transfer system (Bio-Rad, Hercules, CA, USA). The membrane was blocked in TBST (TBS (150 mM NaCl, 20 mM Tris-HCl, pH 7.4) and 0.1% (*v*/*v*) Tween 20) containing 3% (*w*/*v*) bovine serum albumin (BSA), incubated with the indicated primary antibodies as follows: cytochrome c (1 : 1000; rabbit monoclonal, Cell Signaling Technology, Beverly, MA, USA), caspase-3 (1 : 1000; rabbit monoclonal, Cell Signaling Technology), poly(ADP-ribose) polymerase (PARP) (1 : 1000; rabbit monoclonal, Cell Signaling Technology), translocase of the outer membrane 20 (TOM20) (1 : 1000; rabbit monoclonal, Cell Signaling Technology), cytochrome c oxidase subunit IV (COX IV) (1 : 1000; rabbit monoclonal, Cell Signaling Technology), Hsp70 (1 : 2500; mouse monoclonal, Enzo Life Sciences, Farmingdale, NY), *β*-actin (1 : 5000; rabbit monoclonal, Novus Biologicals, Littleton, CO, USA), and GAPDH (1 : 1000; mouse monoclonal, Cell Signaling Technology). The secondary antibody (1 : 5000 in blocking buffer) was incubated at room temperature for 2 h before standard enhanced chemiluminescence detection. For densitometric analyses, western blot films were scanned and processed using a LAS 4000 (GE Healthcare, Chicago, IL, USA) imaging system. ImageJ software (https://imagej.nih.gov/ij/) was used for densitometric measurement of the specific bands of interest. Values were normalized to *β*-actin or GAPDH.

### 2.7. Assessment of Mitochondrial Mass

MitoTracker Green FM (M7514, Thermo Fisher Scientific) is a mitochondrion-selective probe that becomes fluorescent in the lipid environment of mitochondria. MitoTracker Green FM contains a thiol-reactive chloromethyl moiety, resulting in stable peptide and protein conjugates after accumulation in mitochondria, thus allowing estimation of mitochondrial mass in live cells [25]. HL-60 cells were seeded into a 96-well plate at a density of 5 × 10^4^ cells per well. Then, cisplatin and CSE were added to the cells. At 24 h, HL-60 cells were incubated at 37°C with 500 nM MitoTracker Green FM in phosphate-buffered saline (PBS) for 45 min and washed twice with PBS. A SpectraMax M5 microplate reader (Molecular Devices, USA) was used to detect the fluorescence emission at 516 nm in response to alternating 490 nm excitation.

### 2.8. Mouse Bone Marrow Colony-Forming Unit-Granulocyte Macrophage (CFU-GM) Assay

Eight-week-old male Balb/c mice were obtained from BioLASCO (Taipei City, Taiwan). The mice were maintained on pelleted food and water *ad libitum* and housed in controlled environmental conditions (22 ± 1°C and a 12 h light/dark cycle). The protocol for the animal study was approved by the Institutional Animal Care and Use Committee of Chung-Hsing University (protocol no. 108-107).

After the mice were sacrificed, femurs were dissected. Bone marrow cells were flushed with IMDM, counted, and kept in a melting ice bath until use. A total of 1 × 10^6^ bone marrow cells was resuspended in IMDM supplemented with 20% fetal calf serum, 10% conditioned medium of recombinant mouse interleukin-3 (rmIL-3), 10% citrate bovine plasma, and 1.5 mg/mL CaCl_2_. The cultures were incubated with CSE (10, 100, and 500 *μ*g/mL) for 7 days at 37°C in a humidified atmosphere of 5% CO_2_ and 95% air. The formation of colonies was observed by microscopy. Colonies of at least 50 cells were scored at 40x magnification.

For cisplatin and CSE experiments, mice were intraperitoneally (i.p.) injected with three doses of cisplatin on days 1-3 and received CSE by oral gavage on days 4-10. The control group received sterile distilled water by oral gavage on days 4-10. The animals were sacrificed on day 10 to perform CFU-GM assay.

### 2.9. Hematopoietic Cell Survival Assay

At sacrifice, whole blood (2 mL) was collected from ICR mouse (BioLASCO, Taipei City, Taiwan) via heart puncture into K2EDTA-containing tubes. The peripheral blood mononuclear cells (PBMCs) were separated from the whole blood diluted 1 : 2 with PBS by gradient centrifugation using Histopaque®-1077 (Sigma-Aldrich). The mixture was centrifuged at 400 ×*g* for 30 min at room temperature. The PBMCs were washed once with PBS and pelleted down; then, red blood cell (RBC) was lysed with 2 mL of RBC lysis buffer (1x) for 4 min and stop the reaction by adding 8 mL of 1x PBS. Finally, cells were spun down and resuspended in RPMI-1640 medium supplemented with 10% (*v*/*v*) FBS, HEPES (20 nM), 2-mercaptoethanol, penicillin (100 U/mL), and streptomycin (100 *μ*g/mL).

For measurement of CSE effect, PBMCs (2 × 10^4^ cells per well) were seeded into a clear bottom black-well plate or a round bottom plate for 24 h at 37°C in a humidified 5% CO_2_ atmosphere and treated with cisplatin (4 *μ*M) and CSE (25, 50, and 100 *μ*g/mL) for 7 days. The viable cells were determined by CyQuant Direct Cell Proliferation Assay (C35012, Invitrogen) according to the manufacturer's protocol. CyQuant reagent dye was added in a volume of 100 *μ*L, incubated for 2 h protected from light at room temperature. Fluorescence intensity at 480 nm excitation and 535 nm emission was measured using a microplate spectrophotometer.

### 2.10. Animals and Treatments

Male BALB/c mice (7-10 weeks old) were purchased from BioLASCO (Taipei City, Taiwan). Mice were housed under specific pathogen-free conditions in the Biomedical Research Animal Laboratory, Industrial Technology Research Institute. All protocols were approved by the Institutional Animal Care and Use Committee and conducted accordingly to the Guide for the Care and Use of Laboratory Animals (protocol no. 2015-032).

**Figure 1:**
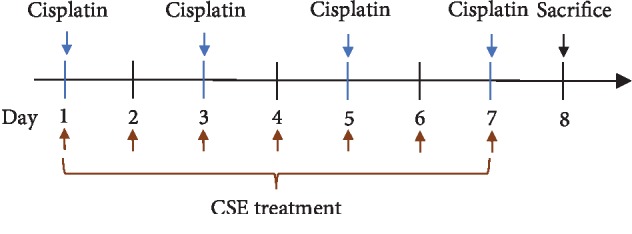
Study scheme of cisplatin and CSE treatment. Mice were divided into 3 groups: (i) control group: 10 mL/kg/day of 5% Dextrose by i.p. injection on days 1, 3, 5, and 7; distilled water (10 mL/kg/day) by oral gavage on days 1-7. (ii) Cisplatin group: cisplatin (5 mg/kg/day) by i.p. injection on days 1, 3, 5, and 7; distilled water (10 mL/kg/day) by oral gavage on days 1-7. (iii) CSE groups: cisplatin (5 mg/kg/day) by i.p. injection on days 1, 3, 5, and 7; CSE (9.6 mL/kg/day) by oral gavage on days 1-7.

Mice were randomly divided into 3 groups (*n* = 8 per group) for cisplatin experiments and treated with CSE (starting from day 1) as shown in Figure 1. Mice were intraperitoneally (i.p.) injected with 4 doses of cisplatin (5 mg/kg/day) on days 1, 3, 5, and 7, while the control group received 10 mL/kg of 5% Dextrose on days 1, 3, 5, and 7. For CSE treatment, mice received CSE at 9.6 mL/kg/day by oral gavage on days 1-7. The control and vehicle groups received sterile distilled water at 10 mL/kg/day by oral gavage on days 1-7. One day after last CSE administration, all animals were euthanized by CO_2_ inhalation.

### 2.11. Body Weight Evaluation

Body weights of individual mice were measured periodically during the study. Weight gain was calculated by subtracting the weight on a given day from the initial weight. Percentage change in body weight of mice was evaluated.

### 2.12. Bone Marrow Cell Preparations

Mouse bone marrow cells were isolated from the femoral bones of BALB/c mice as described previously with slight modifications [26]. In brief, femoral bones were removed from CO_2_-euthanized mice under sterile conditions and immersed in ice-cold Hank's balanced salt solution (HBSS) containing 100 U/mL penicillin and 100 *μ*g/mL streptomycin (all from Thermo Fisher Scientific). Both epiphyses of the femurs were removed with sterile scissors, and bone marrow cells were collected by strongly flushing the diaphysis with ice-cold HBSS using a 1 mL syringe. RBCs were lysed using a 0.9% (*w*/*v*) NH_4_Cl solution (Stemcell Technologies, Cambridge, MA, USA). After washing with HBSS, bone marrow cells were resuspended in RPMI-1640 media containing 2% FBS, 100 U/mL penicillin, and 100 *μ*g/mL streptomycin. Viable cells were counted using the trypan blue exclusion method.

### 2.13. Splenocyte Preparations

Mice were euthanized by CO_2_ asphyxiation, and spleens were immediately removed aseptically. The tissues were grounded through a 70 *μ*m pore-sized meshed cell strainer (Thermo Fisher Scientific) into ice-cold RPMI-1640 media containing 100 U/mL penicillin and 100 *μ*g/mL streptomycin (all from Thermo Fisher Scientific). After a 1,600 rpm centrifugation for 5 min at 4°C, red blood cell lysis was carried out for 5 sec in 450 *μ*L of sterile distilled water and neutralized by adding 50 *μ*L of 10x PBS (pH 7.4). Cells were centrifuged at 1,600 rpm for 3 min at room temperature and resuspended in RPMI-1640 media containing 2% FBS, 100 U/mL penicillin, and 100 *μ*g/mL streptomycin. Viable cells were counted using the trypan blue exclusion method.

### 2.14. CFU-GM Assay in Bone Marrow and Splenocytes

Viable bone marrow cells and splenocytes were homogeneously dispersed in the MethoCult™ GF M3534 medium (Stemcell Technologies) at densities of 1 × 10^4^/mL and 2 × 10^4^/mL, respectively. Cells were plated in 35 mm culture dishes (Stemcell Technologies) and incubated at 37°C in a humidified atmosphere of 5% CO_2_. Following a 9-day (bone marrow cells) and 13-day (splenocytes) incubation, the number of CFU-GM with colonies consisting of more than 50 cells was manually counted under an inverted microscope (BX51, Olympus, Tokyo, Japan). The average colony number of the quadruplicated dishes per group was represented for each specimen.

### 2.15. Histological Analysis

The sternums and spleens were dissected from mice and fixed at 10% neutral buffered formalin (Thermo Fisher Scientific) for 3-5 days at room temperature. Bone decalcification was achieved by immersing the samples in Surgipath Decalcifier I (Leica Microsystems, Richmond, IL, USA) for 2 h at room temperature. The decalcified bone and spleen samples were then dehydrated using a tissue processor (Histo-Tek VP1; Sakura Finetek Japan, Tokyo, Japan) and embedded in paraffin (Nippon Seiro, Tokyo, Japan) according to the standard procedure. Longitudinal 5 *μ*m thick sections were obtained, collected on microscope slides (Muto Pure Chemicals, Tokyo, Japan), and stained with hematoxylin and eosin (H&E). Images were obtained by a BX51 microscope (Olympus, Tokyo, Japan) and acquired with cellSens Standard 1.6 imaging software (Olympus, Tokyo, Japan).

### 2.16. Immunohistochemistry Analysis

Immunohistochemistry was performed on histological sections of formalin-fixed paraffin-embedded spleen samples by using the BOND-MAX Fully Automated IHC and ISH Staining System and Bond Polymer Refine Detection System (Leica Biosystems, Wetzlar, Germany) as per the manufacturer's protocol with proprietary reagents. Briefly, slides were deparaffinized on the automated system with Bond Dewax Solution (Leica Biosystems). Antigen retrieval method was used in sodium citrate buffer (pH 6) for samples for 30 min. The rat primary monoclonal antibody that reacts to mouse CD11b (1 : 100, LifeSpan BioSciences, Seattle, WA, USA) was used at a 1 : 100 concentration in 10% animal serum in tris-buffered saline/0.09% Proclin 950 and incubated for 25 min. The secondary antibodies used were linker rabbit anti-rat IgG (H&L) (1 : 100, ImmunoReagents, Raleigh, NC, USA) for 8 min and polymer goat anti-rabbit-HRP-IgG (1 : 100, ImmunoReagents) for 8 min. The reaction was developed with a diaminobenzidine (DAB) substrate kit (Leica Biosystems) for 3 min. Sections were counterstained with hematoxylin for 3 min. The tissue slides were mounted with gum and a cover glass. Images were obtained by a BX51 microscope (Olympus, Tokyo, Japan), acquired with cellSens Standard imaging 1.6 software (Olympus, Tokyo, Japan).

### 2.17. Statistical Analysis

All data are expressed as mean ± the standard error of the mean (SEM). Statistical differences between groups were analyzed by one-way analysis of variance (ANOVA) followed by Dunnett's multiple comparisons test. The analyses were performed using statistical software R (version 3.4.1; GraphPad Software, Inc., La Jolla, CA, USA). Results were considered statistically significant for *P* values less than 0.05.

## 3. Results

### 3.1. CSE Protects Myeloid Cells against Cisplatin-Induced Cytotoxicity

**Figure 2:**
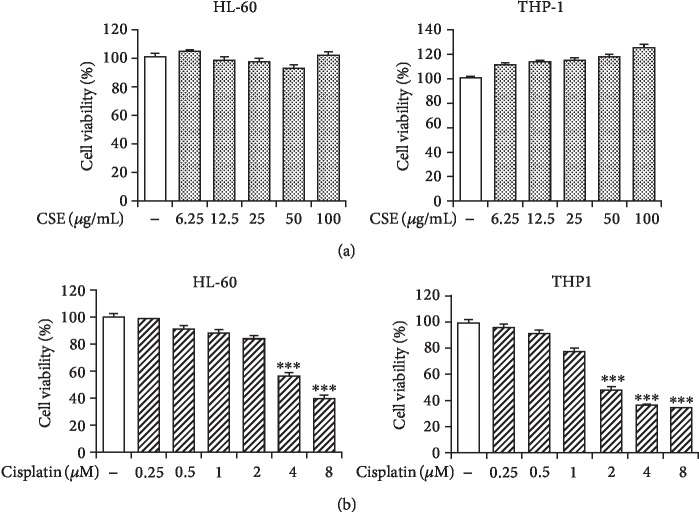
Effect of CSE and cisplatin on myeloid cell proliferation. The HL-60 and THP-1 were cultured in CSE (a) or cisplatin (b) at indicated concentrations for 72 h. Cell viability was measured using the Alamar Blue assay. Data are expressed as mean ± SEM (*n* = 3). Differences among groups were analyzed by one-way ANOVA and post hoc Dunnett's test. ^∗∗∗^*P* < 0.001 versus the untreated control group.

HL-60 and THP-1 have been commonly used as cellular models to study protective effect against chemotoxicity to myeloid cells [27–31]. To determine the dose response to CSE and cisplatin, varying concentrations of CSE (6.25-100 *μ*g/mL) and cisplatin (0.25-8 *μ*M) were preliminarily tested on myeloid cell lines for 72 h. Alamar Blue assay revealed no significant cytotoxicity was observed in HL-60 and THP-1 cells with 6.25-100 *μ*g/mL CSE (Figure 2(a)). However, cisplatin elicited significant reduction of cell viability at 4 *μ*M and 2 *μ*M in HL-60 (57.1 ± 0.6%, *P* < 0.001) and THP-1 (49.1 ± 0.7%, *P* < 0.001) cells, respectively (Figure 2(b)). Thus, cisplatin at a concentration of 4 *μ*M or below and 100 *μ*g/mL of CSE were taken for further experiments.

**Figure 3:**
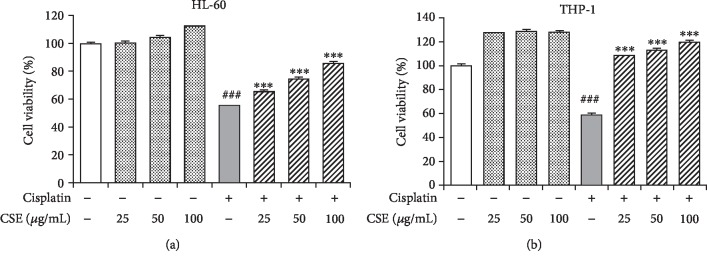
CSE protects cells from cisplatin-induced cytotoxicity. The HL-60 (a) and THP-1 (b) were cultured in cisplatin at 4 *μ*M and 2 *μ*M, respectively, with or without CSE (25, 50, and 100 *μ*g/mL) for 72 h. Cell viability was measured using the Alamar Blue assay. Data are expressed as mean ± SEM (*n* = 3). Differences among groups were analyzed by one-way ANOVA and post hoc Dunnett's test. ^###^*P* < 0.001 versus the untreated control group; ^∗∗∗^*P* < 0.001 versus the cisplatin group.

For studying cytoprotective effects of CSE, we initially treated myeloid cells with CSE and cisplatin for 72 h. Incubation with cisplatin significantly reduced cell viability in HL-60 cells (*P* < 0.001, Figure 3(a)) and THP-1 cells (*P* < 0.001, Figure 3(b)); however, this decrease was greatly attenuated by CSE (25-100 *μ*g/mL) treatment in a dose-dependent manner (*P* < 0.001, Figures 3(a) and 3(b)). At the maximal dose of 100 *μ*g/mL, CSE showed no cytotoxic activity alone after 3 days of incubation. Thus, these results indicate that CSE could rescue cisplatin-evoked cytotoxicity.

### 3.2. CSE Inhibits Cisplatin-Induced Apoptosis in Cancer Myeloid Cells

**Figure 4:**
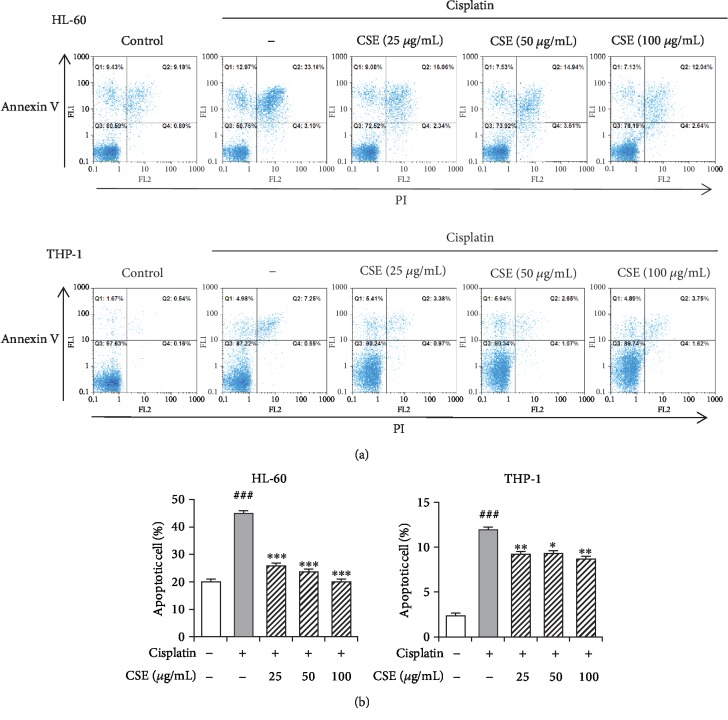
CSE reduces apoptosis in cisplatin-treated cells. The HL-60 and THP-1 cells were cultured with 4 *μ*M and 2 *μ*M cisplatin, respectively, with or without CSE (25, 50, and 100 *μ*g/mL) for 72 h. After staining with Annexin V and propidium iodide, the apoptotic cells were analyzed by flow cytometry (a), and the percentages of early apoptotic (top left quadrant) and late apoptotic (top right quadrant) cells were calculated (b). Data are expressed as mean ± SEM (*n* = 3). Differences among groups were analyzed by one-way ANOVA and post hoc Dunnett's test. ^###^*P* < 0.05 versus the untreated control group; ^∗^*P* < 0.05, ^∗∗^*P* < 0.01, and ^∗∗∗^*P* < 0.001 versus the cisplatin group.

Since cisplatin induces apoptosis in HL-60 cells [32–34], we were interested to know if CSE can block cisplatin-induced apoptosis by staining with PI and Annexin V. An insult with cisplatin led to pronounced apoptosis in HL-60 (45.2 ± 0.8%, *P* < 0.001) and THP-1 (11.9 ± 0.4%, *P* < 0.001) (Figures 4(a) and 4(b)). However, CSE treatment (25-100 *μ*g/mL) reduced the apoptotic HL-60 and THP-1 cells to approximately 19.7-26.1% and 8.6-9.4%, respectively (Figures 4(a) and 4(b)). These results suggest that CSE inhibits cisplatin-induced apoptosis in myeloid cells.

### 3.3. CSE Attenuates Cisplatin-Induced Caspase-3-Dependent Apoptosis in HL-60 Cells

**Figure 5:**
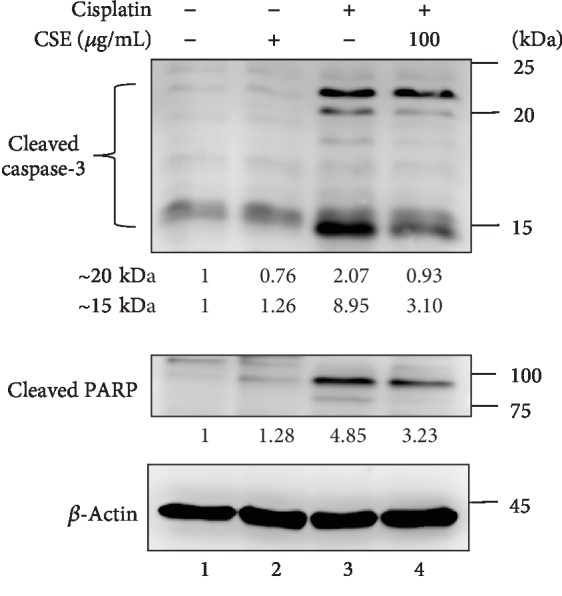
CSE diminishes cisplatin-induced caspase-3 and PARP activation. HL-60 cells were treated with CSE (100 *μ*g/mL) and/or cisplatin (4 *μ*M) for 48 h. Whole cell lysates were collected and subject to western blot analysis for the indicated proteins. Representative images were shown for cleaved caspase-3 and cleaved PARP. *β*-Actin was used as an internal control. Quantification of blots was performed by using ImageJ, and the fold changes to untreated controls are presented.

To gain insight into the mechanism of CSE on decreasing cisplatin-induced apoptosis, levels of caspase-3 and PARP were evaluated using immunoblot. As shown in Figure 5, treating HL-60 cells with cisplatin (4 *μ*M) increased cleaved caspase-3 and cleaved PARP (Lane 3), which was reversed by the administration of CSE at 100 *μ*g/mL (Lane 4). These results support the evidence that CSE rescues HL-60 cells from cisplatin-induced apoptosis through the caspase-3-dependent pathway.

### 3.4. CSE Mitigates Cisplatin-Evoked Caspase-3 Activation through the Mitochondrial Pathway

**Figure 6:**
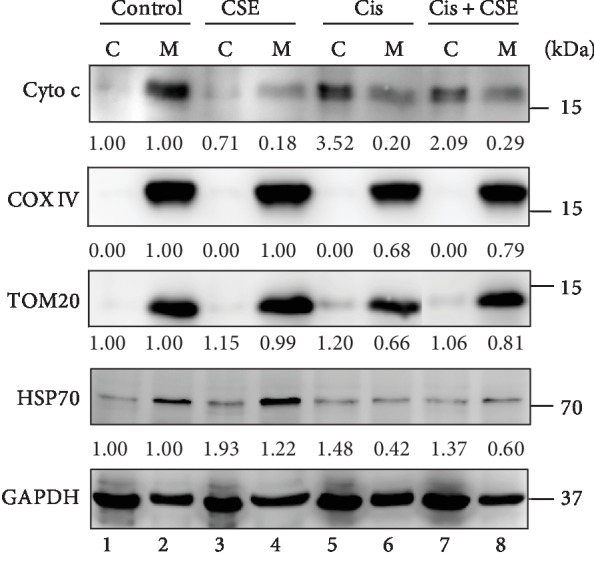
CSE alleviates cisplatin-elicited mitochondrial damage. HL-60 cells were treated with CSE (100 *μ*g/mL) and/or cisplatin (4 *μ*M) for 48 h. The expression levels of cytochrome c, TOM20, COX IV, and HSP70 were examined in the cytosolic and mitochondrial fractions by western blot analysis. GAPDH was used as an internal control. Quantification of blots was performed by using ImageJ, and the fold changes to untreated control are presented. C: cytosolic fraction; M: mitochondrial fraction.

**Figure 7:**
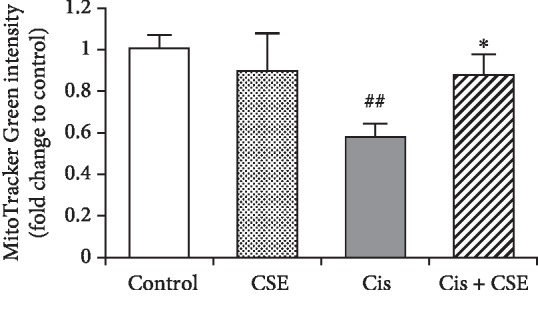
CSE reserves mitochondrial mass. HL-60 cells were pretreated with 100 *μ*M CSE for 30 min followed by incubation with 4 *μ*M of cisplatin for 24 h. Cells were stained with 500 nM of MitoTracker Green FM for 45 min, and the fluorescence of cell extracts was measured using a fluorescence plate reader. Data are the means ± SEM (*n* = 3). ^##^*P* < 0.01 versus the untreated control group; ^∗^*P* < 0.05 versus the cisplatin group.

Considering caspase-3 is activated by proapoptotic molecules such as cytochrome c released from mitochondria [35], we measured cytochrome c in the cytosolic and mitochondrial fractions prepared from myeloid cells. As shown in Figure 6, increasing amounts of cytochrome c were detected in the cytosol from HL-60 cells treated with cisplatin (Lane 5), whereas the corresponding mitochondrial fractions from the same cells showed a depletion of cytochrome c (Lane 6). However, the elevated cytochrome c level in cytosol was reduced upon 100 *μ*g/mL CSE treatment (Lane 7, Figure 6). Similarly, CSE increased the levels of COX IV and TOM20 in cisplatin-treated mitochondrial fractions, though to a somewhat smaller extent (Lane 8, Figure 6). To further substantiate these observations, we used a mitochondrial-specific dye (MitoTracker Green FM) that binds mitochondrial membrane independently of the membrane potential, and thus staining intensity has been considered an index of mitochondrial mass [36]. Notably, the decrease in MitoTracker Green staining induced by cisplatin was restored in HL-60 cells treated with CSE (Figure 7). These results support the idea that reduction of cisplatin-triggered caspase-3 activation by CSE is possibly mediated by the mitochondrial pathway.

### 3.5. CSE Protects Normal Myeloid Cells against Cisplatin-Induced Toxicity

**Figure 8:**
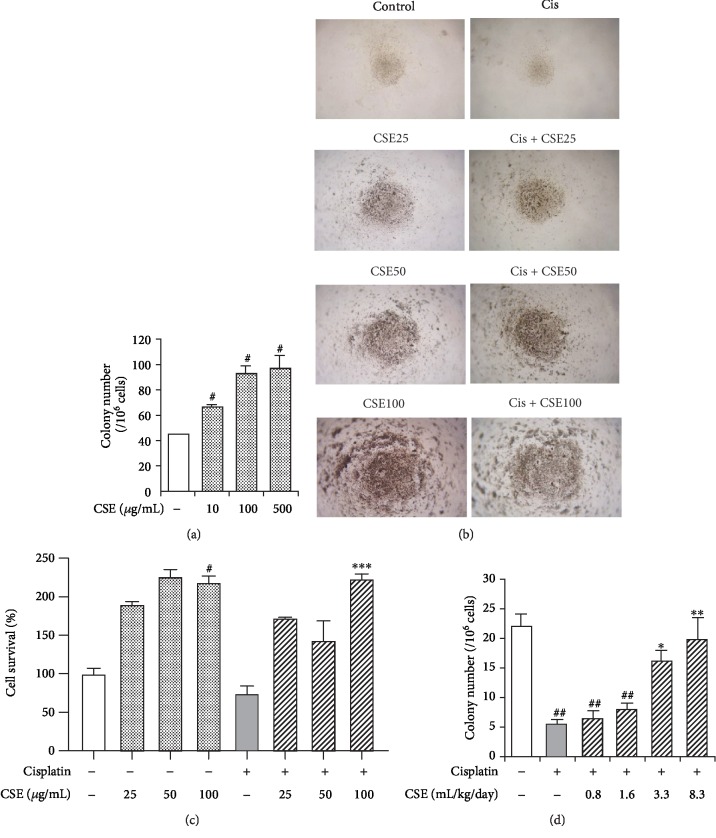
CSE protects normal myeloid cells from cisplatin-induced toxicity. (a) Mouse bone marrow cells were incubated with CSE (10, 100, and 500 *μ*g/mL) for 7 days. The colony number of CFU-GM per bone marrow was counted. (b) Mouse PBMCs were cultured in cisplatin at 4 *μ*M, with or without CSE (25, 50, and 100 *μ*g/mL) for 7 days. Cell morphology was recorded by a bright-field phase-contrast microscopy. (c) Cell viability was measured using the CyQuant direct cell proliferation assay. (d) Mice were i.p. injected with three doses of cisplatin (5 mg/kg/day) for 3 days and received CSE at indicated concentrations by oral gavage for 7 days. Bone marrow cells were cultured in growth medium for 7 days. The colony number of CFU-GM per bone marrow was counted. Data are expressed as mean ± SEM (*n* = 3). Differences among groups were analyzed by one-way ANOVA and post hoc Dunnett's test. ^#^*P* < 0.05 and ^##^*P* < 0.01 versus the untreated control group. ^∗^*P* < 0.05, ^∗∗^*P* < 0.01, and ^∗∗∗^*P* < 0.001 versus the cisplatin group.

In order to test whether normal myeloid cells could also confer chemoprotection to cisplatin *in vitro*, mouse bone marrow cells first incubated with CSE for 7 days. CFU-GM assay revealed CSE at 10, 100, and 500 *μ*g/mL significantly increased the number of CFU-GM after 7-day treatment (*P* < 0.05, Figure 8(a)). Similarly, CSE alone treatment promoted mouse PBMC proliferation by the CyQuant Direct assay (Figures 8(b) and 8(c)). A slight reduction in proliferation rate was observed in 4 *μ*M cisplatin-treated PBMCs. Nevertheless, cell survival was increased by CSE (25, 50, and 100 *μ*g/mL) compared to cisplatin exposure (Figure 8(c)).

Next, to investigate whether CSE possesses beneficial effects on normal myeloid cells *in vivo*, mice were administered cisplatin (5 mg/kg) for 3 days and treated with CSE for 7 days. CFU-GM assay revealed CSE at 10, 100, and 500 *μ*g/mL increased the number of CFU-GM after 7-day incubation (Figure 8(d)). The number of CFU-GM was significantly (*P* < 0.01) reduced in cisplatin-treated mice (Figure 8(d)). Strikingly, this decrease was reversed by CSE treatment at 0.8-8.3 mL/kg. These results highlight the role of CSE in the protection of normal myeloid cells from cisplatin.

### 3.6. CSE Ameliorates Cisplatin-Induced Bone Marrow Hypocellularity

**Figure 9:**
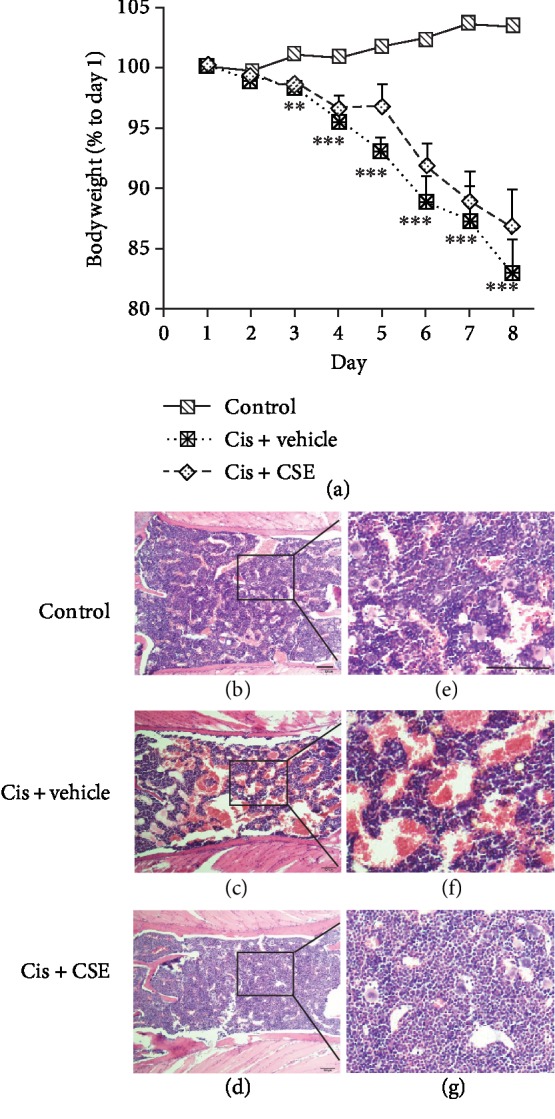
Effect of CSE on mice receiving cisplatin. (a) Body weight changes after cisplatin treatment. Mice were administered with CSE (9.6 mL/kg/day) or distilled water (CIS+vehicle) by gavage for 7 days in the i.p. injection of cisplatin (5 mg/kg/day) on days 1, 3, 5, and 7. Data are represented as mean ± SEM (*n* = 8 per group). The significance of the data was analyzed by one-way ANOVA with post hoc Dunnett's test. ^∗∗^*P* < 0.01 and ^∗∗∗^*P* < 0.001 versus the control group. (b–g) CSE ameliorates cisplatin-induced myelotoxicity. H&E stained sections of formalin-fixed, paraffin embedded sternums were observed at 100x (b–d) and 400x (e–g) magnification; bar, 100 *μ*m. Shown are representative images (*n* = 5‐8 per group).

To evaluate further the effect of chemoprotection in a clinically relevant setting, we analyzed whether CSE treatment affects body weight or bone marrow in mice receiving cisplatin. Compared to the control group, a steady decrease in body weight was observed after cisplatin administration (Figure 9(a)). The decline in body weight was pronounced on days 3-7 of cisplatin treatment. CSE at 9.6 mL/kg showed no significant effects on weight loss induced by cisplatin. Treatment of CSE did not have a significant effect on weight loss induced by cisplatin, indicating that body weight may not be a sensitive enough measure for chemoprotection of CSE against cisplatin. In contrary, 9.6 mL/kg of CSE (Figures 9(d) and 9(g)) ameliorated the marked reduction of bone marrow cells (hypocellularity) induced by cisplatin treatment (Figures 9(c) and 9(f)). These findings suggest that CSE is capable to protect bone marrow from cisplatin-evoked toxicity.

### 3.7. CSE Restores Hematopoietic Progenitor Cells after Cisplatin Treatment

**Figure 10:**
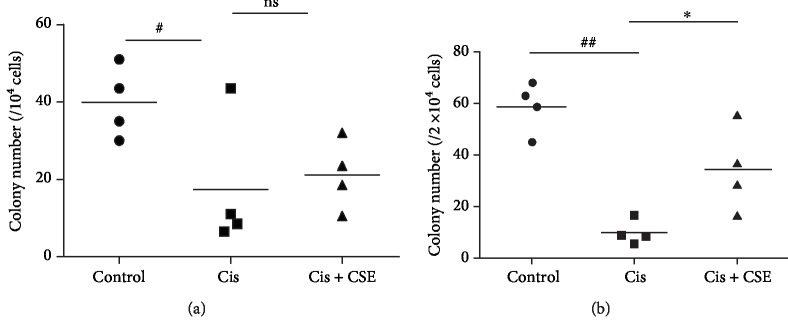
CSE restores hematopoietic progenitors in mice after cisplatin administration. One day after the last cisplatin injection, bone marrow (a) and spleen (b) cells were cultured for respective 9 and 13 days, and the colony number of CFU-GM per bone marrow and spleen was counted. Data are represented as mean ± SEM (*n* = 4 per group). The significance of the data was analyzed by one-way ANOVA with post hoc Dunnett's test. ^#^*P* < 0.05 and ^##^*P* < 0.01 versus the control group; ^∗^*P* < 0.05 and ns (not significant) versus the cisplatin group.

**Figure 11:**
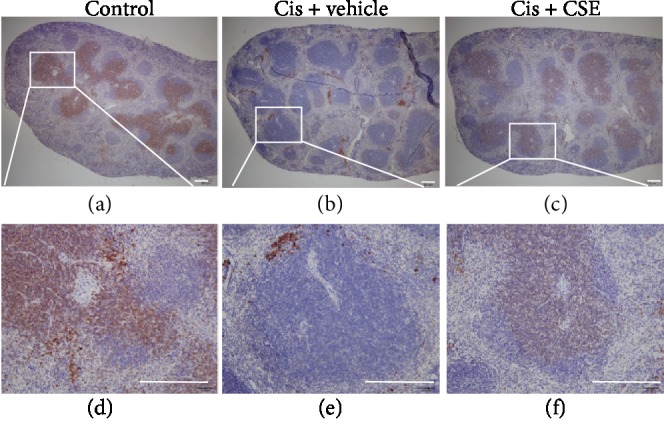
CSE recovers CD11b levels in cisplatin-treated mice. Cisplatin was administered at 5 mg/kg/day for 4 doses in the absence or presence of CSE (9.6 mL/kg/day) for 7 days. Immunohistochemical analysis was conducted with anti-CD11b to analyze spleen and observed by a microscope at 40x (a–c) and 200x (d–f) magnification; bar, 50 *μ*m. Shown are representative images (*n* = 5‐8 per group).

The CFU-GM assay has been well recognized as a substitute to experimental animals to predict myelotoxicity in humans [37]. Because bone marrow hypocellularity might result from inadequate growth of hematopoietic progenitors in cisplatin-treated mice, we examined the *in vitro* clonogenic potential of committed myeloid progenitors. As shown in Figure 10(a), the number of colonies from hematopoietic progenitors from cisplatin-administered mice was markedly reduced by approximately 56% (*P* < 0.05), as compared with controls. In contrast, CSE treatment increased CFU-GM activity in bone marrow, but differences were not statistically significant (Figure 10(a)). Likewise, the number of colonies obtained from cisplatin-treated splenocytes was also severely depressed for the myeloid assay when compared with controls (*P* < 0.01; Figure 10(b)). However, CSE treatment led to higher CFU-GM activity in the spleen compared to cisplatin alone (*P* < 0.05; Figure 10(b)). Correspondingly, CSE at 9.6 mL/kg greatly restored the granulocyte/macrophage biomarker CD11b expression in splenocytes exposed to cisplatin (Figures 11(c) and 11(f)). Thus, these data suggest that CSE could protect hematopoietic progenitors in response to the toxicity of cisplatin.

## 4. Discussion

Here, we show that the extract of *Chlorella sorokiniana* provides chemoprotective effects against cisplatin *in vitro* and *in vivo*. In this study, we reveal that CSE abrogates cisplatin-induced cytotoxicity by reducing cell death (Figures 3 and 4), suppressing apoptosis signaling (Figure 5), and preventing mitochondrial damage (Figures 6 and 7) in cancer myeloid cells. CSE also confers protection from cisplatin in mouse bone marrow cells and PBMCs (Figure 8), as well as restores hematopoietic progenitor cells after cisplatin treatment in the spleen (Figures 10 and 11). These results demonstrate CSE's positive effect on protecting myeloid cells from the insult of cisplatin. Additionally, the beneficial effects observed for bone marrow (Figure 9) from mice receiving cisplatin support CSE's relevance and potential therapeutic value in treating cisplatin-evoked toxicity.

Cisplatin chemotherapy has been a mainstay of cancer treatment since approved by the FDA in 1978 [2] for a broad range of cancers [3]. Despite being efficacious on damaging tumor cells via cross-linking with DNA and induction of apoptosis [4], cisplatin is associated with several side effects resulted from hepatotoxicity, nephrotoxicity, ototoxicity, myelotoxicity, and gastrointestinal toxicity [5]. Consequently, these cumulative and irreversible toxicities reduce the potential options for cisplatin as a future treatment on relapse. Hence, its use is limited in terms of dose and duration of treatment, with subsequent decreased tumor control and survival, and ultimately interfering with patient safety and quality of life.

In our *in vitro* model of cisplatin-induced toxicity in myeloid cells, we observed that cisplatin treatment markedly increased the number of apoptotic cells that was reversed by CSE treatment. Earlier studies have pointed out that cisplatin induces apoptosis in HL-60 cells through BCL2 downregulation and activation of BCL2L12 expression [33], oxidative stress, and inhibition of cell cycle progression [34]. Also, cisplatin activated the intrinsic pathway of apoptosis through alteration of the mitochondrial membrane potential, release of cytochrome c, and upregulation of caspase-3 activity in acute promyelocytic leukemia (APL) and human T leukemia cells [38]. In agreement with the previous reports, we found cisplatin-evoked caspase-3 activation coincided with the reduction of mitochondrial content in HL-60 cells. Notably, not only did CSE diminish the levels of cleaved caspase-3 and cleaved PARP, but it also decreased the release of cytochrome c to the cytosolic fractions. On top of that, CSE increased the levels of COX IV, TOM20, and HSP70 in mitochondrial fractions, as well as restored mitochondrial mass in cisplatin-treated cells.

Caspase-3 plays a central role in the execution of the apoptotic program [39] and is primarily responsible for the cleavage of PARP during cell death [40]. Indeed, PARP cleavage serves as a marker of cells undergoing apoptosis by preventing futile repair of DNA strand breaks and essentially inactivates the enzyme to incapably respond to DNA strand breaks [41]. Cisplatin binds with high affinity to nuclear DNA and can physically interact with several cytoplasmic nucleophiles, including mitochondrial DNA (mtDNA) as well as multiple mitochondrial and extramitochondrial proteins [42–46]. It is well accepted that these lesions mediate cisplatin's cytotoxic effect.

Mitochondria are the powerhouses of the cell [47]. In addition to ATP generation, mitochondrial electron transport chain is a major cellular source of reactive oxygen species (ROS) (estimated at approximately 90%), mainly H_2_O_2_ from complex I, II, and III [47–50]. Oxidative stress plays a role in the pathogenesis of cisplatin-induced dose-limiting toxicities, and mitochondrial-dependent ROS response enhances the cytotoxic effect caused by nuclear DNA damage [51]. Release of cytochrome c from mitochondria to cytosol causes mitochondrial damage and dysfunction during apoptosis [52]. Besides, the chaperone protein HSP70 has been shown to suppress the mitochondrial release of cytochrome c [53, 54] and cooperates with HSP90 to inhibit cytochrome c-mediated caspase activation [55, 56], thereby halting further caspase activation. The present study demonstrated that HSP70 was elevated by CSE treatment in the mitochondrial fraction of HL-60 cells exposed to cisplatin. These results are supported by previous reports showing reduction of HSP70 enhances cisplatin-induced apoptosis in HGC-27 gastric cancer cells and A529 lung adenocarcinoma cells, as well as accumulation of HSP70 inhibits heat shock-induced apoptosis in HL-60 cells [57–59]. In relation to these findings, it is of interest that we observed that cisplatin-induced release of cytochrome c from mitochondria and compromised mitochondrial function were reversed by CSE. We propose that CSE-mediated rescue of mitochondria to HL-60 reverses the cytotoxic effects of cisplatin, thereby facilitating cell survival.

The improvement in mitochondrial function and survival of myeloid cells in the presence of CSE is likely not limited to the prevention of cell death. Cisplatin is known to generate myelotoxicity [60–62]. However, when we treated the mice with CSE while administration of cisplatin, which elicited hypocellularity of bone marrow, we observed the positive effect of CSE on the restoration of bone marrow *in vivo*. Hematological toxicity such as leukopenia and anemia occurred in approximately half of cisplatin-treated patients with lung cancer and advanced ovarian cancer [63]. Hitherto, hematopoietic growth factors (HGFs) like recombinant granulocyte-colony-stimulating factor (G-CSF) and erythropoietin are first-line choices for the treatment of patients with chemotherapy-induced myelosuppression [60]. Nevertheless, the use of HGFs has been impeded by their high costs and their own side effects including myalgia, bone pain, pulmonary infiltrates, rash, and thrombophlebitis [63]. Furthermore, the potential of G-CSF to promote tumor growth by enhancing neovascularization in a tumor raised a critical safety issue of G-CSF in cancer patients [64]. Therefore, the development of efficient and safer therapeutics or preventives are still needed for the management of cancer patients.

Previous reports have demonstrated natural products are beneficial in cisplatin-induced myelotoxicity in animal models, such as vetiver oil (Java) [65], olive, and olive oil [66]. In the current study, the *in vivo* impact of CSE treatment on cisplatin-induced toxicity was evaluated. Consistent with our *in vitro* findings, cisplatin caused remarkable bone marrow hypocellularity; however, CSE at 9.6 mL/kg preserved bone marrow cellularity (Figures 9(d) and 9(g)). In the non-tumor-bearing host, cisplatin treatment might induce acute hematotoxic injury that leads to stimulation of G-CSF, the major regulator of neutrophilic granulocytes and to rebound leukocytosis [67]. Indeed, G-CSF in combination with IL-1*α* has been found to synergistically enhance recovery of primitive hematopoietic cells in mice exposed to 5-fluorouracil [68]. Thus, we further addressed whether CSE could affect colony formation in bone marrow and spleen after cisplatin treatment. As shown in Figure 10(a), CSE showed a slight tendency to promote hematopoietic progenitor cell CFU-GM activity in bone marrow from mice receiving cisplatin, which is consistent with the fact that bone marrow CFU-GM content is directly linked to recovery of peripheral blood cells [69]. Furthermore, elevated CFU-GM activity (Figure 10(b)) and CD11b levels (Figures 11(c) and 11(f)) in the spleen were also found in CSE-treated mice upon cisplatin exposure. Taken together, these results indicate that CSE exerts a protective role in cisplatin-induced myelotoxicity along with hematopoietic damage.

## 5. Conclusions

**Figure 12:**
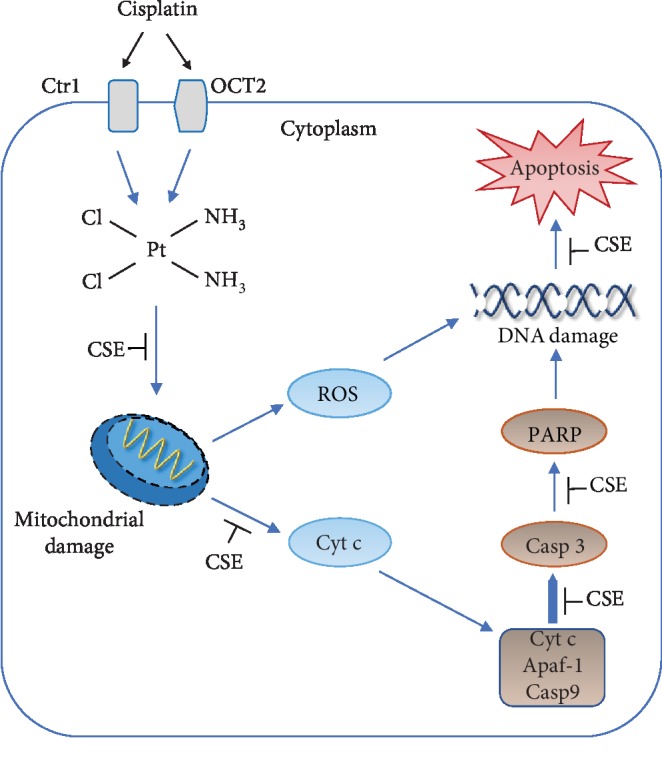
Proposed molecular mechanism for the action of CSE on cisplatin toxicity in human promyelocytic cells. Cisplatin treatment results in mitochondrial damage, release of cytochrome c from mitochondria, activation of caspase-3 and PARP, and apoptosis in HL-60 cells. Upon cisplatin exposure, CSE can preserve mitochondrial mass, reduce cytochrome c release, suppress caspase-3 and PARP activation, and consequently promote cell survival. Ctr1: copper transporter 1; OCT2: organic cation transporter 2; Cyt c: cytochrome c; Casp 3: caspase-3; Casp 9: caspase-9.

In summary, we found that combining CSE administration with cisplatin produced protective effects against bone marrow toxicity, probably through suppression of apoptosis via a mitochondrial-dependent caspase activation pathway (Figure 12). To the best of our knowledge, this is the first study to examine the beneficial effects of *Chlorella sorokiniana* extract in myelosuppression after cisplatin treatment in normal mice. Therefore, this study promises the use of *Chlorella sorokiniana* as a chemoprotective agent but necessitates further experimental (tumor model) and clinical studies to validate our preliminary findings.

## Data Availability

The data that support the findings of this study are available from the corresponding author, Shu-Fang Wen, upon reasonable request.

## Abbreviations

ANOVA: Analysis of variance
APL: Acute promyelocytic leukemia
BSA: Bovine serum albumin
CFU-GM: Colony-forming unit-granulocyte macrophage
CSE: *Chlorella sorokiniana* extract
DAB: Diaminobenzidine
FBS: Fetal bovine serum
G-CSF: Granulocyte-colony-stimulating factor
HBSS: Hank's balanced salt solution
H&E: Hematoxylin and eosin
HGFs: Hematopoietic growth factors
IMDM: Iscove's Modified Dulbecco's Medium
IP: Intraperitoneally
mtDNA: Mitochondrial DNA
PBMCs: Peripheral blood mononuclear cells
PBS: Phosphate-buffered saline
PMSF: Phenylmethylsulfonyl fluoride
RBC: Red blood cell
RIPA: Radioimmunoprecipitation assay
RPMI: Roswell Park Memorial Institute
SEM: Standard error of the mean
TBS: Tris-buffered saline.
